# Tumor classification of gastrointestinal liver metastases using CT-based radiomics and deep learning

**DOI:** 10.1186/s40644-023-00612-4

**Published:** 2023-10-05

**Authors:** Hishan Tharmaseelan, Abhinay K. Vellala, Alexander Hertel, Fabian Tollens, Lukas T. Rotkopf, Johann Rink, Piotr Woźnicki, Isabelle Ayx, Sönke Bartling, Dominik Nörenberg, Stefan O. Schoenberg, Matthias F. Froelich

**Affiliations:** 1 Department of Radiology and Nuclear Medicine, University Medical Center Mannheim, Heidelberg University, Theodor-Kutzer-Ufer 1-3, 68167 Mannheim, Germany; 2 German Cancer Research Center, E010 Radiology, Im Neuenheimer Feld 280, 69120 Heidelberg, Germany

**Keywords:** Deep learning, Radiomics, Machine learning, Metastases, Gastrointestinal

## Abstract

**Objectives:**

The goal of this study is to demonstrate the performance of radiomics and CNN-based classifiers in determining the primary origin of gastrointestinal liver metastases for visually indistinguishable lesions.

**Methods:**

In this retrospective, IRB-approved study, 31 pancreatic cancer patients with 861 lesions (median age [IQR]: 65.39 [56.87, 75.08], 48.4% male) and 47 colorectal cancer patients with 435 lesions (median age [IQR]: 65.79 [56.99, 74.62], 63.8% male) were enrolled. A pretrained nnU-Net performed automated segmentation of 1296 liver lesions. Radiomics features for each lesion were extracted using pyradiomics. The performance of several radiomics-based machine-learning classifiers was investigated for the lesions and compared to an image-based deep-learning approach using a DenseNet-121. The performance was evaluated by AUC/ROC analysis.

**Results:**

The radiomics-based K-nearest neighbor classifier showed the best performance on an independent test set with AUC values of 0.87 and an accuracy of 0.67. In comparison, the image-based DenseNet-121-classifier reached an AUC of 0.80 and an accuracy of 0.83.

**Conclusions:**

CT-based radiomics and deep learning can distinguish the etiology of liver metastases from gastrointestinal primary tumors. Compared to deep learning, radiomics based models showed a varying generalizability in distinguishing liver metastases from colorectal cancer and pancreatic adenocarcinoma.

**Supplementary Information:**

The online version contains supplementary material available at 10.1186/s40644-023-00612-4.

## Introduction

Cancer is one of the leading causes of death worldwide, with metastatic disease being one of the main reasons for mortality. The liver is one of the most common sites for metastatic spread [1]. Due to the strong portal venous influx into the liver and their high prevalence, colorectal and pancreatic cancer are frequent origins of hematogenous liver metastases [2]. For colorectal cancer, 22% of the patients show distant metastases at the time of diagnosis. For pancreatic cancer, the number of patients showing metastases at the time of diagnosis is even higher with 52%. Despite modern targeted therapies, 5-year survival of patients with metastatic colorectal cancer (mCRC) (14.7%) and metastatic pancreatic adenocarcinoma (mPA) (3.0%) is limited in the advanced stages of the disease [3, 4].

Clinically, the differential diagnosis of liver metastases in patients with multiple primary tumors can be a challenging task. In the case of solitary primary tumors, the underlying tumoral entity is regularly determined from clinical information of a known primary tumor. However, biopsies of (multiple) liver metastases in a patient are rarely acquired in clinical practice due to the disproportional invasiveness. As a result, differentiating tumor primary by quantitative analysis of non-invasive imaging methods would provide a clinical advantage.

Machine learning (ML) can be applied to molecular genetic data to identify the primary tumor [5]. In the case of multiple primary tumors, imaging-based “virtual biopsy” could provide similar clinical benefits, cost and time reduction for confirmation of the diagnosis, and support as a first signpost in planning further interventions [6, 7].

Earlier studies have shown that radiomics features and deep learning features can help to assess interlesional variability, underlying entities [8–10], and response [11–13]. However, the potential methodology ranges from radiomics features [14], often combined with ML-classifiers [15], and applications of convolutional neural networks (CNN) [16]. First approaches to classify liver lesions have been performed using radiomics [17] and CNN [18]. However, their comparative performance in determining liver metastases primary has not been analyzed head-to-head.

Therefore, this benchmark study aims to assess the performance of radiomics- and CNN-based classifiers on a test dataset to determine the primary cancer origin of gastrointestinal tumors by characterizing liver metastases of colorectal and pancreatic adenocarcinoma.

## Materials and methods

### Patient collective and imaging protocols

**Fig. 1:**
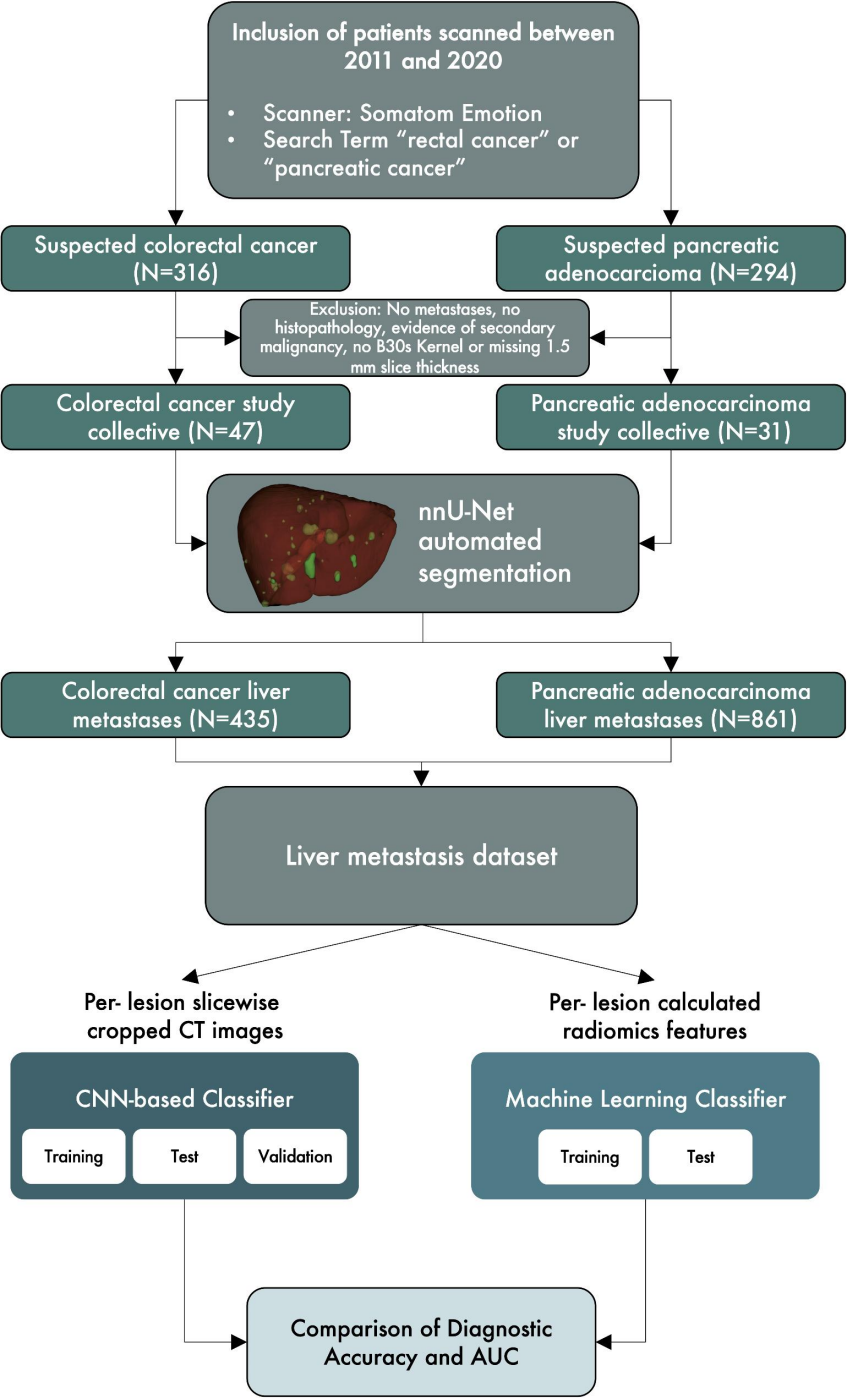
Consort flow diagram showing the search terms, cohort selection criteria and structure of following analysis

Patients with hypoattenuating liver metastases in mCRC and mPA that were examined in a 16-slice CT-scanner (Siemens Somatom Emotion, Siemens Healthcare GmbH, Erlangen, Germany) in our institution between 2011 and 2020 were identified retrospectively by the search terms “rectal cancer” and “pancreatic cancer.“ Only scans that were reconstructed in B30s Kernel with 1.5 mm slice thickness in axial orientation and acquired in portal venous contrast enhancement phase (60 s delay, 90 ml intravenous Imeron® (Bracco Imaging, Milan, Italy), 2.5 ml/s flow) were included. Based on our inclusion criteria, 47 mCRC patients and 31 mPA patients were included. In the mCRC population, 36.2% of the patients were female and had a median age of 64. Compared to that in the mPA collective, 51.6% of the patients were female and had a median age of 65.39. The patient characteristics for both groups are summarized in Table 1. The study protocol is summarized as a CONSORT diagram in Fig. 1.

**Table 1:** Patient characteristics. Median and IQR

		Colorectal cancer cohort	Pancreatic cancer cohort
**n**		47	31
**Age (median [IQR])**		65.79 [56.99, 74.62]	65.39 [56.87, 75.08]
**Sex (%)**	F	17 (36.2%)	16 (51.6%)
	M	30 (63.8%)	15 (48.4%)
**T-Stage (%)**	T1	2 (4.3%)	1 (3.2%)
	T2	4 (8.5%)	3 (9.7%)
	T3	24 (51.1%)	6 (19.4%)
	T4	15 (31.9%)	19 (61.3%)
	Tx	2 (4.3%)	2 (6.5%)
**N-Stage (%)**	N0	8 (17.0%)	6 (19.4%)
	N1	18 (38.3%)	11 (35.5%)
	N2	20 (42.6%)	14 (45.2%)
	Nx	1 (2.1%)	
**M-Stage (%)**	M1	47 (100.0%)	31 (100%)
**Liver lesions**	Number	435	861
	Per patient	9.25	27.77
	Mean HU	45.89	44.93
**Liver parenchyma**	Liver tumor burden	9.8%	6.7%
	Mean HU	91.13	86.35

### Liver and lesion segmentation

To minimize the effect of inter-rater variability and create comparable results, a manual approach was chosen [19, 20]. Liver and liver lesions were segmented fully automated using the Applied Computer Vision Lab (ACVL) nnUNet pretrained liver segmentation model [21]. The created segmentations were corrected, if necessary, by a medical student (H.T., two years of experience in radiological image segmentation). Afterward, they were reviewed by a clinical radiologist (M.F.F. with more than four years of experience in oncologic imaging). The metastases segmentation mask was split into single lesion masks using 3DSlicer (version 4.11.20210226) [22]. Lesions smaller than 0.5 cm were considered too small to characterize and excluded. The liver tumor burden, defined as the ratio of metastasis to total liver voxel volume, was estimated by the automated segmentation.

### Split into train, (validation), and test dataset

To evaluate the model’s performance, for both approaches eight patients with four from each group (mPA and mCRC) were randomly selected without stratification as an independent test set. The metastases image slices and radiomics signatures of these patients were not used during the training process. For the DenseNet-121 the dataset of cropped metastases CTs was randomly split into train, validation, and test sets in a ratio of 68/5/8 patients. If a patient had multiple liver metastases, all were only included in one set.

### Radiomics-based classifiers

Radiomics features were extracted from the original images without filtering for patients with mPA and mCRC for each liver lesion using pyradiomics (version 3.0.1) [23]. Corresponding settings can be found in the supplementary material S1. First-order, 2D, and 3D shape features, neighboring gray tone difference (NGTDM), gray level co-occurrence matrix (GLCM), gray level run length matrix (GLRLM), gray level size zone (GLSZM), and gray level dependence matrix (GLDM) features were extracted. Following, the features were selected by applying a Pearson Correlation Coefficient (PCC) threshold of 0.6 for redundancy reduction. To identify the important features for the differentiation of metastases by primary, permutation-based feature importance was calculated using a Random Forest (RF) classifier. To account for imbalances in the input dataset, the synthetic minority over-sampling technique (SMOTE) using the python package imblearn (version 0.9.0) was performed on the training set. Random undersampling was used to investigate possible distortion of the input data by SMOTE. Standardization was applied to both the train and test set before analysis.

Several classification algorithms were implemented for the radiomics dataset: XG Boost (XGB), Random Forest (RF), Support Vector Machine (SVM), K-SVM, K-nearest neighbor, Logistic Regression, Naive Bayes, and Decision Tree. Hyperparameter tuning was performed if applicable to achieve maximum performance. Results from the test dataset were generated to compare lesion-wise performance.

### Image-based CNN classifiers

#### Preprocessing

The clinically diagnosed primary tumor for each patient was assumed as the ground truth for the corresponding liver lesions. To achieve a high degree of confidence, patients with multiple primary tumors were not enrolled. Automatically created segmentation masks were used to blacken the area surrounding the metastases, to only focus on the lesions. Following, the lesions were windowed in an abdominal window (window width of 330/window level of 10), cropped, and exported as images with a size of 224 × 224. Input images were augmented by zooming, shearing, rotation, and width shift and contained the entire lesion along with its borders.

#### Model definition and training

**Fig. 2:**
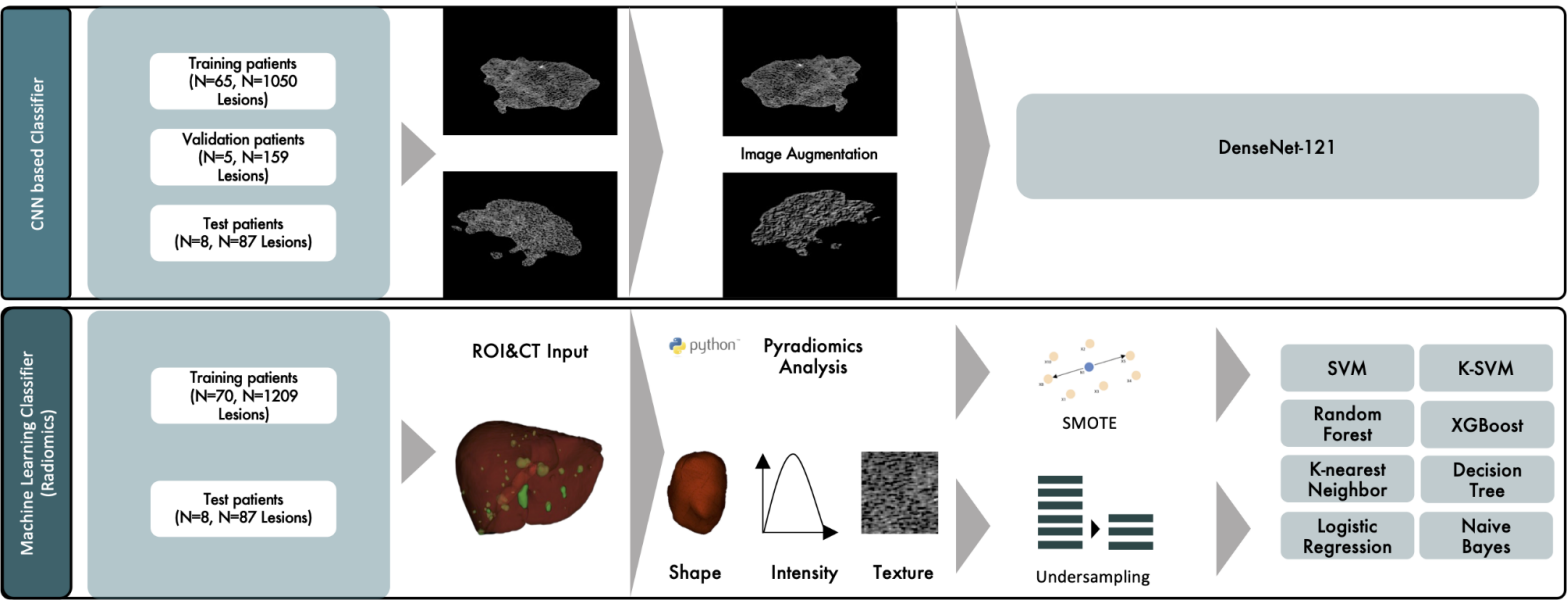
Technical pipeline for radiomics- and Densenet-121-based image slice analysis displaying used models and structure

**Fig. 3:**
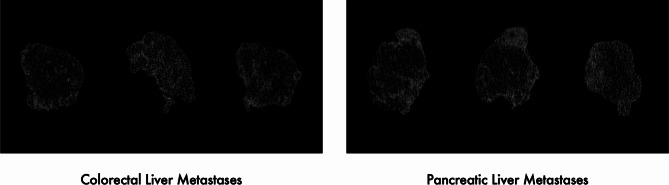
Three example slices of visually indistinguishable liver metastases from each colorectal cancer and pancreatic cancer cohort

The model was trained from scratch using a DenseNet-121 for analysis, and single metastasis slices were used as an input. DenseNet-121 is a dense convolutional neural network algorithm with a depth of 121 layers [24]. The network for supervised learning was implemented in Pytorch. The analysis workflow is summarized in Fig. 2. Example lesions for colorectal and pancreatic liver metastases are displayed in Fig. 3. A detailed description of the model and training settings can be found in supplementary material S2.

#### Lesion-wise comparison

To evaluate the performance on individual lesions, the slice-wise results were cumulated lesion-wise. The cumulated model outputs were classified as pancreatic or colorectal based on a cutoff value of 0.5.

## Results

### Comparison of tumoral distribution patterns

The following segmentation correction and lesion separation resulted in a total of 861 lesions in the mPA group compared to 435 lesions in the mCRC group. For mCRC, patients showed fewer but larger metastases (a mean tumor burden of 9.8% and 9.25 metastases per patient). Patients with mPA had more but smaller metastases (mean tumor burden of 6.7% and an average of 27.77 metastases per patient).

### Radiomics-based classifiers

The PCC threshold of 0.6 resulted in a reduction of 78.1% from 105 to 23 features. The resulting features were ranked by permutation-based importance and listed in Table 2 (all results in S3). Predominantly first order and grayscale texture features were identified as the most important. On the test set from all classifiers (XGB, RF, SVM, K-SVM, K-nearest neighbor, Logistic Regression, Naive Bayes, and Decision Tree), the K-nearest neighbour classifier showed the best performance with an AUC of 0.87 and an accuracy of 0.67 (Table 3) for the differentiation between colorectal and pancreatic liver metastases. Differing number of lesions (n = 435 colorectal liver metastases versus n = 861 pancreatic liver metastases) has led to a large class imbalance. In comparison to SMOTE, undersampling has shown a different but overall comparable performance in terms of accuracy and AUC.

**Table 2:** 10 most important extracted radiomics features, ordered by importance

	Feature	Permutation importance
1	original_firstorder_90Percentile	0.053969
2	original_firstorder_Mean	0.048406
3	original_glszm_GrayLevelNonUniformityNormalized	0.040987
4	original_glcm_Correlation	0.038470
5	original_ngtdm_Complexity	0.037156
6	original_glszm_GrayLevelVariance	0.036443
7	original_glcm_Imc1	0.036336
8	original_firstorder_10Percentile	0.035691
9	original_shape_Flatness	0.035509
10	original_gldm_HighGrayLevelEmphasis	0.034498

### Imaging-based classifiers

After training with the training and validation cohort, the model generalized well on the independent test set and achieved similar results with an AUC of 0.80 and an accuracy of 0.83. Radiomics classifiers outperformed the imaging-based DenseNet-121. Results for the performance of both classifiers can be found in Table 3.

**Table 3:** ML-classifiers and DenseNet-121 performance on the independent test set

		Random undersampling		SMOTE	
Approach	Classifier	AUC	Accuracy	AUC	Accuracy
ML-classifier	XG Boost	0.71	0.84	0.71	0.82
	Random Forest	0.60	0.84	0.62	0.79
	K-means clustering SVM	0.79	0.72	0.79	0.71
	K-nearest neighbour	0.77	0.59	0.87	0.67
	SVM	0.79	0.72	0.79	0.71
	Logistic Regression	0.66	0.53	0.65	0.60
	Gaussian Naive Bayes	0.51	0.52	0.52	0.50
	Decision Tree	0.43	0.36	0.43	0.29
CNN-classifier	DenseNet-121	0.80	0.83	0.80	0.83

### Lesion-wise comparison on the test set

**Fig. 4:**
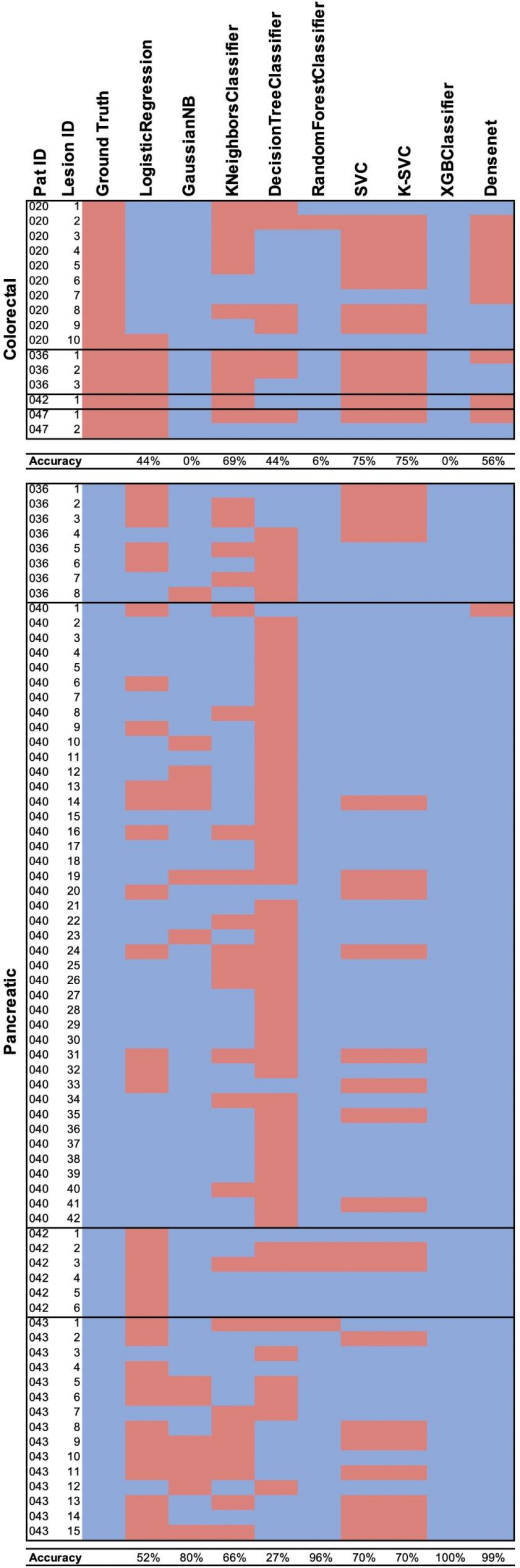
Per-lesion comparison of the model’s performances on test set. Red boxes indicate lesions predicted as colorectal, while blue boxes represent predictions as pancreatic lesions

The lesion-wise comparison on the test set shows a difference in performance of the models based on classes. Both ML- and DL- based classifiers, especially Gaussian Naive Bayes, XGBoost, and Random Forest in average tended to classify lesions rather as pancreatic than colorectal, which led to a better performance in classifying pancreatic cases (Fig. 4). SVM-classifiers showed the best results in detecting colorectal lesions. However, XG Boost and Gaussian NB classifiers could not identify colorectal metastases sufficiently (accuracy: 0%). The model’s performance in terms of classification on an individual lesion level is varying. E.g. in colorectal cancer patient no. 20 KNN-classifier identifies 4/10 lesions as pancreatic and 6/10 as colorectal. This leads to a limited practical value and may be caused by overfitting and/or caused by class imbalances. To address this, larger training datasets could be a possible solution.

## Discussion

This study shows the clinical potential of radiomics-based and deep learning-based approaches to implement AI in the differential diagnosis of visually similar liver lesions by processing information not perceivable to the human eye. It demonstrates the capability of DL/ML-based imaging and radiomics features to identify the underlying tumor entity and may help to establish an automated approach as a “virtual biopsy” of suspicious liver lesions demonstrated for gastrointestinal cancers. In the independent test cohort, radiomics showed a solid performance: Besides first-order features, gray level features, known as imaging biomarkers for tissue texture and heterogeneity, were ranked as important. In comparison, the DenseNet-121 without pre-defined features could also classify the primary tumor based on liver lesion characteristics and showed a comparable performance.

The models showed a high level of variability in predictions within patients of the test set. This could be caused by multiple factors. For instance, prior works have shown an interlesional variability within single patients and one tumoral entity which leads to different radiomics signatures [25]. Such differences exist naturally or can be induced by therapy and may result in the model identifying some lesion types better than others.

The results presented are in line with previous studies, which demonstrated how machine-learning approaches and deep convolutional neural networks could support inexperienced radiologists to differentiate lesions that an experienced radiologist can differ visibly [18]. Our study translates this approach to a topic, where even a high degree of experience may not be associated with a relevant accuracy. The comparison of methods to handle class imbalance have shown that there is an effect of such methods on model performance. Class imbalance in general can be addressed using different methodologies [26]. A possible solution to that is the accurate reporting of used methods.

This work complements and extends previous results as we were able to show how radiomics- and deep learning-based methods can support clinical decision-making as a signpost for visually indistinguishable liver lesions. DL/ML-generated insights may be used in a diagnostic or therapeutic setting to acquire information about prognosis or the targetability of lesions. In this challenging setting, deep learning and ML-radiomics achieved exceptional accuracy. Given the visual indistinguishability of metastatic liver lesions for both assessed gastrointestinal cancer types, this study’s results indicate the substantial potential of quantitative imaging biomarkers to provide information about the tumor biology and cancer origin. Yet, the classifiers cannot give a wholly accurate distinction in every case.

Moreover, tumor tissue arising from a singular primary can show a high degree of heterogeneity, proven by autopsy studies [27], which is a challenge for successful targeted therapies [28]. Therefore, biopsies of solitary lesions may be misleading regarding molecular and histological properties, especially in patients with multiple disseminated liver metastases, and may not reveal mutations as potential targets. As it is known from post-mortem analyses, liver metastases show a notable interlesional molecular variability [29]. Yet, the reliability may be further increased by relying decision-making on quantitative image features and supplementing our approach with clinical features and laboratory biomarkers such as cfDNA and liquid profiling, which have been shown to increase diagnostic accuracy further [9].

This work must be considered in the context of its limitations. Only grayscale images of metastatic liver lesions were used in this retrospective study. The surrounding area of the metastases was not considered to only focus on the features of the lesion. In addition to the tumor tissue itself, quantitative parameters from the tissue surrounding the tumor may provide additional information about the tumor entity [30] and may be investigated in further studies. Due to the inclusion criteria, the results are limited to a relatively homogeneous and small patient collective of comparable scan parameters; other slice thicknesses and kernels need to be assessed to improve the evaluation of performance under these conditions and compare radiomics features’ generalizability on larger datasets. Only portal venous contrast phase CT-scans were used as this is a standard phase in abdominal CT. This study could also be extended to different contrast phases. Furthermore, liver metastases with histologic criteria of adenocarcinoma (of the colon and pancreas) were included in this analysis, while no further histopathological stratification was included. It would be interesting to investigate if radiomics or CNN features may even help differentiate other cancer subtypes (or origins) non-invasively. A combination of both methodologies into one model and training on a larger dataset may boost the power of the model and should be studied in further analyses.

In addition, there are limitations concerning the validation cohort, as no external dataset was used for validation. However, the study has a single-center retrospective design, which may be the foundation for a prospective multi-center study. Yet, this study can be regarded as a proof-of-concept study for the unraveling of textures indistinguishable for the human eye.

## Conclusion

In summary, this study demonstrates the ability of radiomics and image-based deep learning models to distinguish liver metastases based on their primary cancer showing the potential for a non-invasive virtual biopsy and extraction of quantitative imaging biomarkers. In this cohort, radiomics features showed comparable performance to the CNN model in classifying visually indistinguishable liver metastases. This study may be a proof of concept for the often-quoted idea of personalized AI-driven quantitative image diagnostics.

### Electronic supplementary material

Below is the link to the electronic supplementary material.

Supplementary Material 1

## Data Availability

Due to German medical privacy guidelines the datasets used and analysed during the study are available from the corresponding author on reasonable request.
