# Supplementary material for: Tumor classification of gastrointestinal liver metastases using CT-based radiomics and deep learning
Source: Cancer Imaging. 2023 Oct 5;23:95. doi: 10.1186/s40644-023-00612-4 (PMC10557291; doi:10.1186/s40644-023-00612-4)
Supplement: Supplementary file 1 — Supplementary Material 1 [file 40644_2023_612_MOESM1_ESM.docx]

## **Supplemental Material**

**S1: Radiomics settings**

***Extraction parameters***{'minimumROIDimensions': 2, 'minimumROISize': None, 'normalize': False, 'normalizeScale': 1, 'removeOutliers': None, 'resampledPixelSpacing': None, 'interpolator': 'sitkBSpline', 'preCrop': False, 'padDistance': 5, 'distances': [1], 'force2D': False, 'force2Ddimension': 0, 'resegmentRange': None, 'label': 1, 'additionalInfo': True}

Enabled filters: {'Original': {}}

Enabled features: {'firstorder': [], 'glcm': [], 'gldm': [], 'glrlm': [], 'glszm': [], 'ngtdm': [], 'shape': []}

**S2: DenseNet settings**

According to our preprocessing we fitted with an input layer of 224x224x3. Binary cross-entropy loss of the validation set was used to evaluate the model performance. The DenseNet121 was trained for 30 Epochs:

Total params: 7,037,504

For our training, Adaptive Moment Estimation (adam) stochastic gradient function was utilized as an optimization method. Input size 224x224 was selected. The learning rate was set to 10^-6. Early stopping and model checkpoint were used as callbacks with following settings:

early_stop = EarlyStopping(monitor="val_loss",

mode="min",

patience=10)

checkpoint= ModelCheckpoint(filepath= "best_loss_model.hdf5",

monitor="val_loss",

verbose=1,

save_best_only=True,

mode="min")

**S3: All selected features**

|  | **Radiomics feature** | **Permutation importance** |
| --- | --- | --- |
| 1 | original_firstorder_90Percentile | 0.053969 |
| 2 | original_firstorder_Mean | 0.048406 |
| 3 | original_glszm_GrayLevelNonUniformityNormalized | 0.040987 |
| 4 | original_glcm_Correlation | 0.038470 |
| 5 | original_ngtdm_Complexity | 0.037156 |
| 6 | original_glszm_GrayLevelVariance | 0.036443 |
| 7 | original_glcm_Imc1 | 0.036336 |
| 8 | original_firstorder_10Percentile | 0.035691 |
| 9 | original_shape_Flatness | 0.035509 |
| 10 | original_gldm_HighGrayLevelEmphasis | 0.034498 |
| 11 | original_firstorder_RobustMeanAbsoluteDeviation | 0.034493 |
| 12 | original_firstorder_Variance | 0.033540 |
| 13 | original_gldm_LargeDependenceEmphasis | 0.033454 |
| 14 | original_glrlm_RunPercentage | 0.031698 |
| 15 | original_glszm_ZoneEntropy | 0.031164 |
| 16 | original_glszm_SmallAreaEmphasis | 0.030844 |
| 17 | original_glszm_ZonePercentage | 0.030594 |
| 18 | original_glszm_GrayLevelNonUniformity | 0.030586 |
| 19 | original_glszm_LargeAreaHighGrayLevelEmphasis | 0.030232 |
| 20 | original_gldm_DependenceNonUniformity | 0.030051 |
| 21 | original_firstorder_Skewness | 0.028854 |
| 22 | original_glszm_HighGrayLevelZoneEmphasis | 0.028787 |
| 23 | original_shape_LeastAxisLength | 0.028424 |
